# Prediction and analysis of symmetry-raising transitions in anilinium tetra­fluoro­borate

**DOI:** 10.1107/S2052520625009618

**Published:** 2025-11-13

**Authors:** Sam Y. Thompson, Chloe A. Fuller, Samuel J. Page, Andrew J. Bell, John S. O. Evans

**Affiliations:** a Department of Chemistry Durham University Lower Mount Joy, South Road Durham DH1 3LE United Kingdom; b Swiss–Norwegian Beamlines European Synchrotron Radiation Facility Grenoble France; c School of Chemical and Process Engineering University of Leeds Leeds LS2 9JT United Kingdom; University of Geneva, Switzerland

**Keywords:** crystal structure, phase transition, ferroelectricity, dielectric constant, disorder

## Abstract

We report the structural and dielectric characterization of anilinium tetra­fluoro­borate (AnBF_4_), a molecular crystal identified as a candidate for symmetry-raising phase transitions using the *FERROSCOPE* routine. Variable-temperature powder X-ray diffraction and differential scanning calorimetry revealed a sequence of three structural phase transitions between room temperature and 490 K, yielding four polymorphs (A–D), three of which are previously unreported. All structures were solved either by single-crystal X-ray diffraction or *ab initio* from powder diffraction data. Single crystal experiments indicate diffuse scatter in polymorph C which can be related to the structural changes between the different polymorphs. The structural transitions have been rationalized using group–subgroup relationships. Dielectric measurements show a temperature-dependent response consistent with the structural changes, with the A to B transitions associated with a peak in the dielectric constant.

## Abstract 2

Three symmetry-raising phase transitions of anilinium tetrafluoroborate between room temperature and 490 K have been structurally characterized using powder and single-crystal diffraction, dielectric measurements, NMR and group–subgroup relationships.

## 1. Introduction

Symmetry-raising phase transitions are central to understanding a wide range of important solid state properties including ferroelectricity, piezoelectricity, conductivity, magnetism, NLO-switching and others (Ok *et al.*, 2006 ▸; Shi *et al.*, 2016 ▸). Identifying materials with such transitions can allow the prediction of exploitable properties. For example, a ferroelectric switching mechanism can often be inferred from the structural changes needed to go from a polar structure to a non-polar higher-symmetry form. Being able to predict whether a given structure has the potential to undergo a symmetry-raising phase transition is therefore a powerful way to identify new functional materials (Abrahams, 1988 ▸; Abrahams, 2000 ▸; Abrahams, 2008 ▸; Dypvik Sødahl *et al.*, 2023 ▸; Seyedraoufi *et al.*, 2024 ▸; Capillas *et al.*, 2004 ▸).

We have recently developed the program *FERROSCOPE* which can identify molecular crystal structures likely to undergo structural phase transitions (Thompson *et al.*, 2025 ▸). This is done by applying chemically-sensible simplifications to a given structure, followed by a search for pseudosymmetry that suggests proximity to a higher symmetry parent structure (Evans & Thompson, 2025 ▸). Such structures have a high likelihood of undergoing phase transitions. *FERROSCOPE* has been used to search the 1.3 million entries in the Cambridge Structural Database (CSD) and has identified around 17000 such structures.

In this study, we present an experimental investigation of the molecular crystal, anilinium tetra­fluoro­borate (AnBF_4_),
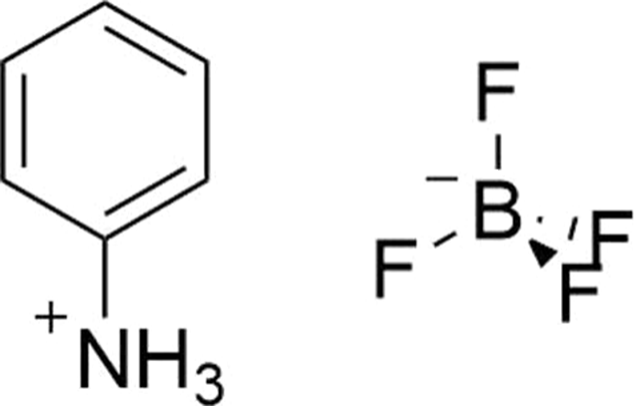
which was identified as being likely to display symmetry-raising phase transitions by *FERROSCOPE*.

At 100 K, AnBF_4_ crystals are monoclinic with polar space group *P*2_1_ (polymorph A, CSD refcode: QEHWEG) (Anderson *et al.*, 2006 ▸). Head-to-tail chains of anilinium ions point parallel to the *c* axis with their molecular planes rotated 27° out of the *bc* plane. Neighbouring chains point in opposite directions and are rotated in the opposite sense. BF_4_^−^ ions are positioned between four anilinium rings, sitting slightly closer to the aminium end of the anilinium due to coulombic interactions. The polarization arises from the collective displacement of BF_4_^−^ anions along the *b* axis.

**Figure 1:**
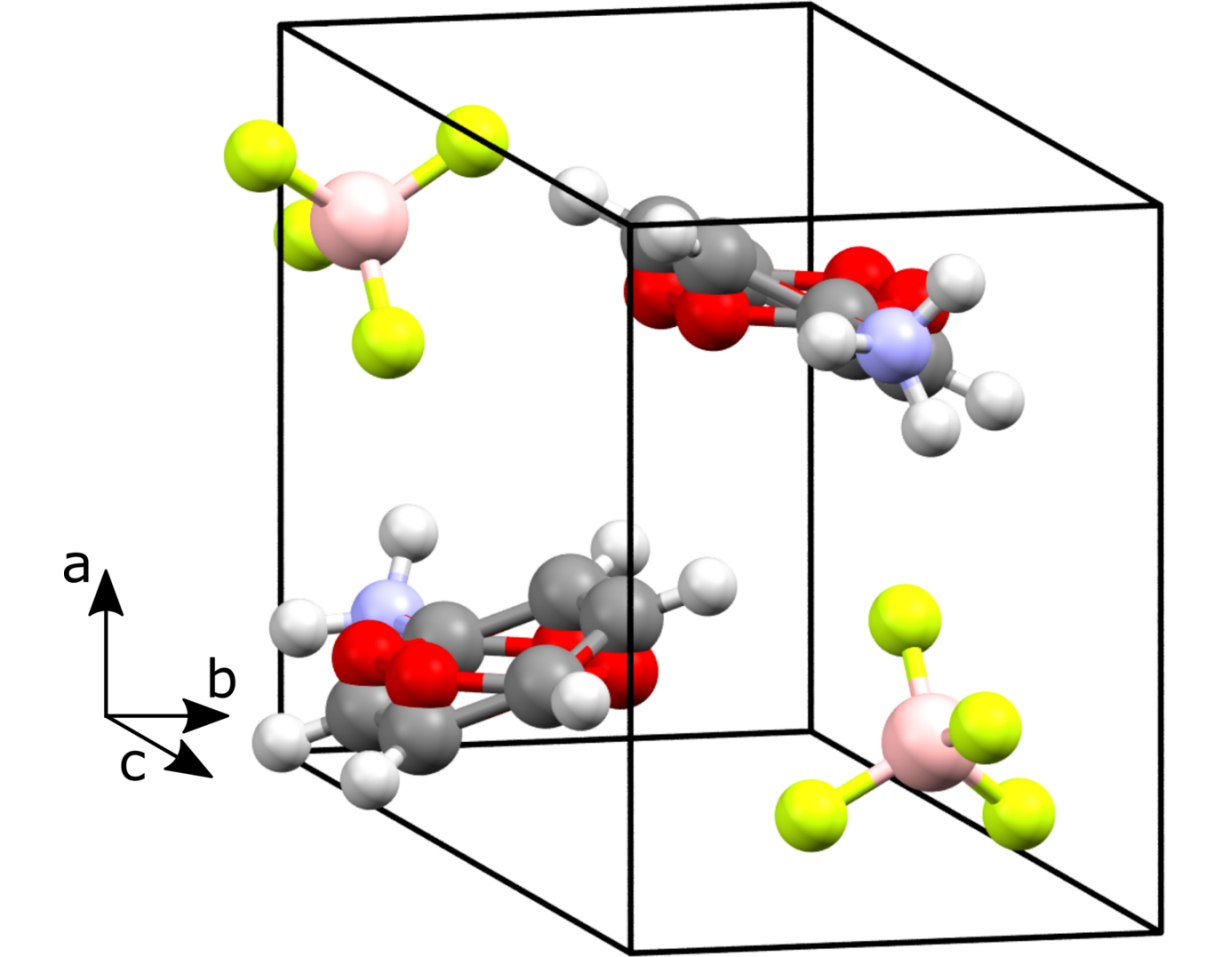
Room-temperature structure of AnBF_4_. The anilinium ring positions of the higher-symmetry *P*2_1_/*m* structure predicted by *FERROSCOPE* are shown in red.

*FERROSCOPE* performs a series of structural modifications on input structures before attempting to identify higher symmetry. These are based on structural changes known to occur in molecular ferroelectrics. For AnBF_4_, the fluorine atoms were removed from the BF_4_^−^ to represent the spherical symmetry these anions adopt at high temperatures due to rotational tumbling. Hydrogen atoms were removed as they provide little additional structural information over the carbon skeleton, while for the –NH_3_ groups, proton removal represents rapid rotation at high temperature. The modified structure was found to have a closely related higher-symmetry structure in *P*2_1_/*m*. The most significant predicted motion is the rotation of the aniliniums into the *bc* plane which creates the new mirror symmetry in the structure (Fig. 1 ▸). The predicted ferroelectric switching mechanism is therefore similar to that observed in 4-(cyano­methyl)­anilinium perchlorate (Cai *et al.*, 2011 ▸).

In this paper we report variable-temperature powder X-ray diffraction (VT-PXRD) experiments to look for this predicted phase transitions. A series of significant changes in the Bragg peaks between 300 K and 490 K indicated the formation of three new polymorphs, labelled B, C and D. Structures were determined for all three either directly from the PXRD data or, where possible, from single-crystal X-ray diffraction (SXRD) data. All new and previously reported structures can be related using a group–subgroup tree allowing their interconversion to be understood.

## 2. Experimental

### 2.1. Synthesis

Anilinium tetra­fluoro­borate was synthesized by adding aniline (1 ml, 11.2 mmol, Merck Life Sciences, ≥99.5%) to water (5 ml). Fluoro­boric acid (1.8 ml, 48 ± 2%wt in water, Aldrich) was then added dropwise over 1 min. The solution was stirred for 10 min, then the solvent was allowed to evaporate over one week, leading to colourless block-shaped crystals. Polycrystalline samples were prepared by gentle grinding of the crystals.

### 2.2. Powder X-ray diffraction

VT-PXRD data were collected using a Bruker D8 ADVANCE Mo *K*α diffractometer, equipped with a LYNXEYE detector and an Oxford Cryosystems Cryostream Plus device. The sample was sealed in a 0.7 mm external diameter borosilicate capillary to a length of 30 mm, and was spun at ten rotations a minute during the measurements. Data were collected between 130 and 490 K with a warming/cooling rate of 20 K h^−1^ and a collection time of 28 min which corresponds to a pattern being recorded every 9.33 K. Structure solution from powder data and Rietveld refinement was performed in the *TOPAS* software package (Coelho, 2018 ▸; Coelho *et al.*, 2011 ▸; Dinnebier *et al.*, 2018 ▸).

### 2.3. Single-crystal X-ray diffraction

SXRD data were collected with a Bruker D8 VENTURE diffractometer (PHOTON III C7 MM CPAD detector, ImS-microsource, focusing mirrors) using Mo *K*α radiation. Sample temperatures were set using an Oxford Cryosystems Cryostream 700+ device. Crystal structures were solved and refined within the *Olex2* software package (Dolomanov *et al.*, 2009 ▸). H atoms were placed in calculated positions and refined in riding mode.

Synchrotron diffraction data were collected at the BM01 end station of the Swiss–Norwegian Beamlines at the ESRF (Dyadkin *et al.*, 2016 ▸). Data were collected at 0.69437 Å with the detector 0.439 m from the crystal. The crystal was rotated 360° about a single axis and frames were collected across an angular range of 0.1° for 0.5 s each. An Oxford Cryosystems Cryostream 700+ was used to keep the crystal at 430 K. Data were reduced and integrated, and scattering planes were reconstructed in *CrysAlis^PRO^*. Resulting diffuse scattering data were interpreted by comparing observed scattering to that calculated from supercell Monte Carlo models constructed in *DISCUS* (Proffen & Neder, 1997 ▸). Details on the simulations are provided in the supporting information.

### 2.4. Differential scanning calorimetry (DSC)

DSC data were collected between 300 and 470 K with a heating rate of 5 K h^−1^ using a TA Instruments DSC2500. AnBF_4_ (7.380 mg) was placed in an aluminium pan and referenced against an empty pan under an N_2_ atmosphere.

### 2.5. Dielectric measurements

Thin pellets (∼0.5 mm, 13 mm diameter) of AnBF_4_ were produced for polarization–electric field measurements and thicker pellets (∼2 mm, 10 mm diameter) for dielectric constant measurements with applied pressures of 10 and 7 tons, respectively. Conductive silver paste was deposited on both flat sides of the pellets and cured at room temperature.

Polarization–electric field measurements were performed by connecting the painted electrodes to a Radiant Technologies Precision LCII ferroelectric tester. A drive voltage of up to 5 kV was applied with switching frequencies of 1, 10 and 100 Hz. Dielectric constant measurements were performed using a Hewlett Packard HP4284 LCR on a sample placed in a furnace. ɛ, ɛ′ and tan δ were recorded at frequencies ranging from 100 Hz to 1 MHz up to 420 K.

### 2.6. Solid-state NMR

^13^C solid-state NMR spectra were recorded at 100.63 MHz using a Bruker Avance III HD spectrometer and a 3.2 mm (rotor outer diameter) magic-angle spinning probe. The spectra were obtained using cross-polarization (CP). A recycle delay of 40 s and a cross polarization contact time of 1 ms were used. Data were acquired at a sample spin-rate of 20 kHz. Spectral referencing was with respect to an external sample of neat tetra­methyl­silane. Temperatures were calibrated against a lead nitrate reference (Beckmann & Dybowski, 2000 ▸). A static measurement and MAS spectra with a spinning rate of 20 kHz were recorded on heating. The temperature offset deviation was modelled with a linear fit which was applied to the set value of the temperature controller for these measurements. The calibration was validated against an ethyl­ene glycol at various temperatures.

Chemical shifts were calculated for the room-temperature structure of AnBF_4_ using density-functional theory in the *CASTEP* package (Clark *et al.*, 2005 ▸) which uses a plane-wave basis set and pseudopotentials to represent core electrons. A plane-wave cut-off energy of 700 eV was applied, and a 2 × 2 × 2 k-point grid offset by (0.25, 0.25, 0.25) was used. The local-density approximation (LDA) (Ceperley & Alder, 1980 ▸; Perdew & Zunger, 1981 ▸) exchange-correlation functional was used. Atomic positions and unit-cell parameters were fixed at the values obtained from the X-ray diffraction data. NMR shielding tensors were computed using the GIPAW method (Pickard & Mauri, 2001 ▸) and the chemical shifts were correlated to the experimental shifts by linear regression resulting in a referencing constant of 164.53 p.p.m. This was applied within *MagresView 2* (Sturniolo *et al.*, 2016 ▸) and Lorentzian peak shapes were generated with a 1.5 p.p.m. peak width.

^19^F solid-state NMR spectra were recorded at 376.50 MHz using a Bruker Avance III HD spectrometer and a 3.2 mm (rotor outer diameter) magic-angle spinning probe. The spectra were obtained using direct polarization with high-power proton decoupling (HPDEC). A recycle delay of 4 s was used. Data were acquired at a sample spin-rate of 20 kHz. Spectral referencing was with respect CFCl_3_.

## 3. Results and discussion

### 3.1. Observations of structural phase transitions in AnBF_4_

**Figure 2:**
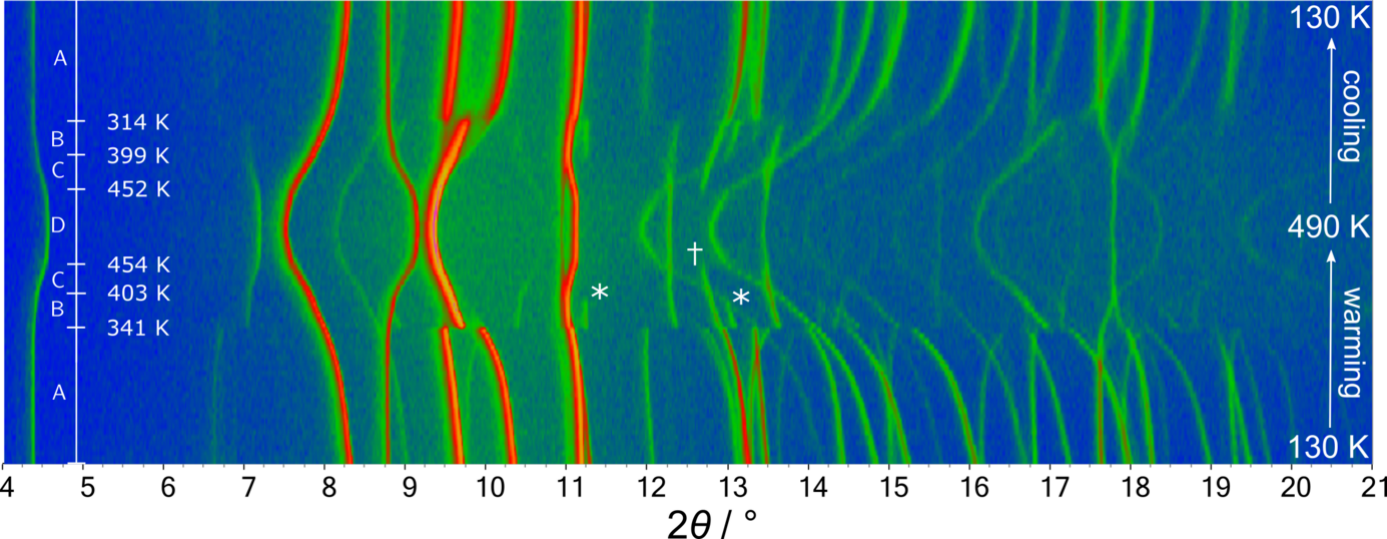
Two-dimensional plot of VT-PXRD data on warming and cooling between 120 and 490 K. An artificial colour map represents the intensities of the data: low intensity is blue and high intensity is orange. Labelled peaks are discussed in the text.

To assess the accessibility of the predicted high-symmetry structure of AnBF_4_, we performed variable-temperature powder X-ray diffraction (VT-PXRD) experiments. Data are shown in Fig. 2 ▸. By inspection, three phase transitions are observed in this temperature range. AnBF_4_ adopts the monoclinic form A when synthesized and retains this structure down to 130 K.

Upon warming, a phase transition occurs at about 341 K. The convergence of *hkl* and *hk**l* reflections, *e.g.* (111) and (111) originally at 9.5° and 10.3° (130 K), suggest this is likely a monoclinic to orthorhombic transition. Two subsequent phase transitions occur at ∼403 K and ∼454 K showed, *inter alia*, by the disappearance of weak peaks in the 2θ range 11° to 13° (marked with *), then loss of the peak marked †. We label the sequence of polymorphs as A (monoclinic, 130 to 341 K), B (orthorhombic, 341 to 403 K), C (orthorhombic, 403 to 454 K) and D (orthorhombic, 454 to >490 K). On cooling, the two higher temperature phase transitions show little hysteresis: their transition temperatures on cooling were ∼452 K and ∼399 K. The lowest temperature transition between polymorphs A and B occurs at 314 K on cooling as opposed to 341 K on warming showing hysteresis and discontinuous character. DSC data measured on AnBF_4_ similarly revealed three peaks in the temperature range 300 to 490 K (Fig. S1). These gave phase transition temperatures on warming/cooling between polymorphs A and B (336/321 K), B and C (400/396 K), and C and D (448/445 K). The smaller hysteresis of the second two transitions and the less dramatic changes in the diffraction data suggest that two higher temperature transitions are weakly discontinuous whereas the first is strongly discontinuous.

**Figure 3:**
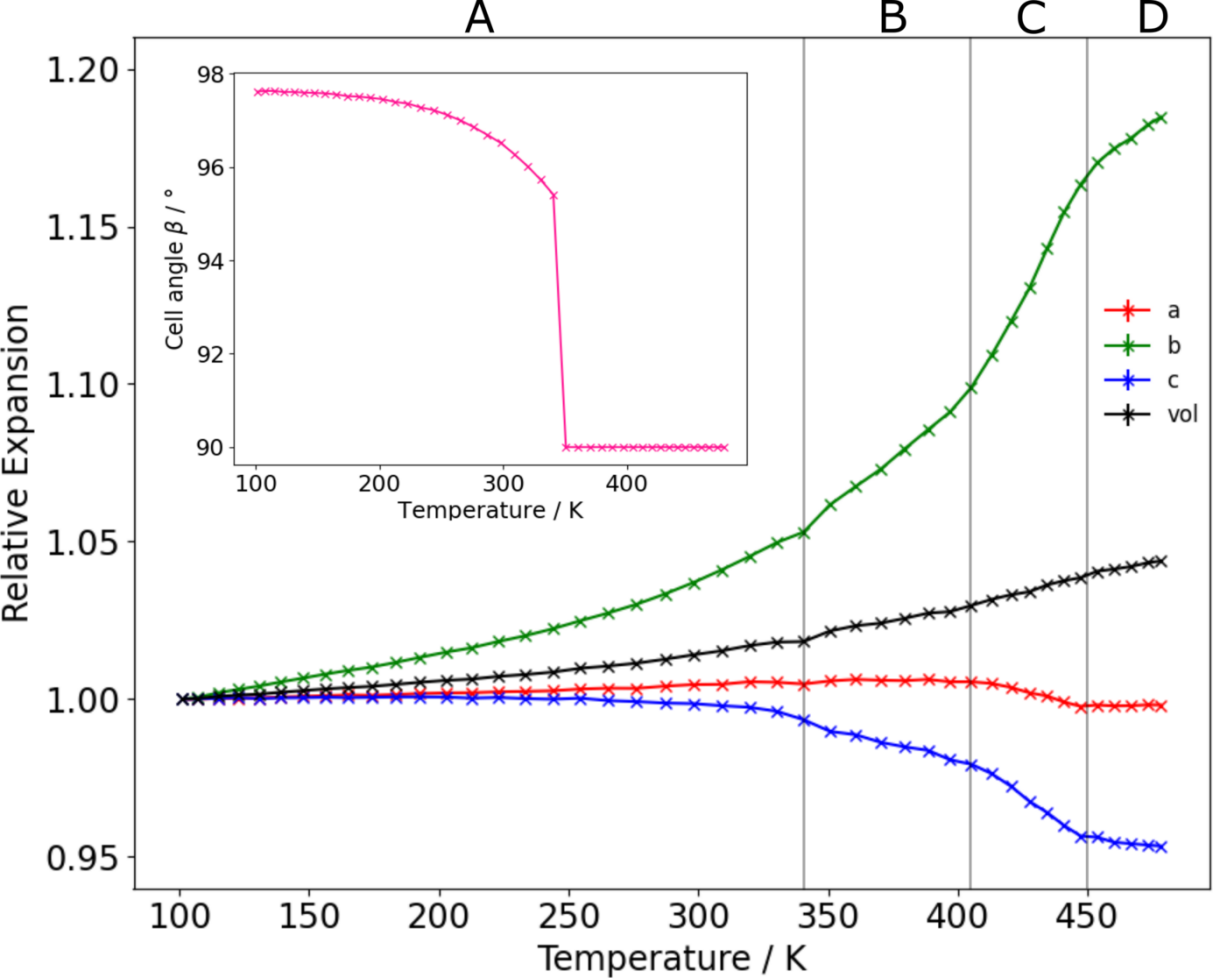
Relative changes in unit-cell parameters of AnBF_4_ extracted from sequential Rietveld fits to VT-PXRD data on warming. Inset: the change in the β angle with increasing temperature.

The reversible behaviours seen in Fig. 2 ▸ and in the DSC measurements show that no change in sample composition occurs during any of these transitions. Unit-cell parameters were extracted from the PXRD data and are plotted in Fig. 3 ▸. They show four discrete regions of thermal expansion behaviour, one for each phase. Generally, the cell contracts along the *a* and *c* axes upon warming but this is countered by the large expansion along the *b* axis leading to net positive thermal expansion.

### 3.2. Crystal structure of polymorph A

**Figure 4:**
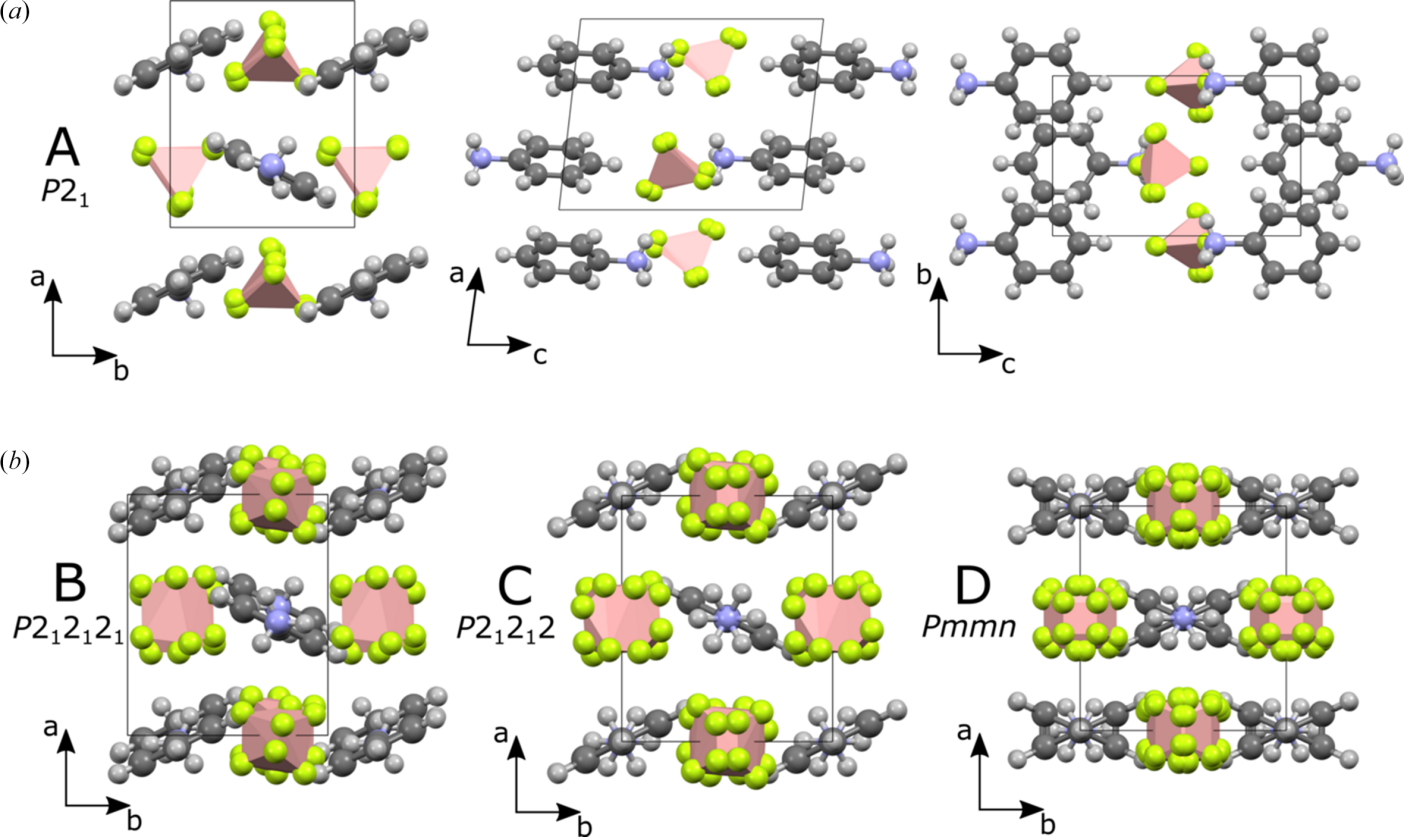
(*a*) Crystal structure of polymorph A at room temperature viewed along each axis. (*b*) Crystal structures of the new higher temperature polymorphs of B, C and D viewed along the *c* axis.

AnBF_4_ was previously reported in the literature by Anderson *et al.* (2006 ▸). It was found to crystallize in space group *P*2_1_ at 100 K with unit-cell parameters *a* = 7.3752 (16) Å, *b* = 5.7860 (9) Å, *c* = 9.3866 (19) Å, β = 97.620 (11)° from SXRD data. This was confirmed by our own SXRD experiment which gave unit-cell parameters of *a* = 7.3672 (9) Å, *b* = 5.9633 (7) Å, *c* = 9.3181 (11) Å, β = 96.520 (4)° at 290 K and a similar structural model [Fig. 4 ▸(*a*)]. Our diffraction data are best modelled with the BF_4_^−^ anions rotationally disordered across two equally occupied sites, whereas Anderson *et al.* (2006 ▸) used an ordered model. The difference is likely due to temperature-induced disorder being less prevalent at 100 K. There are N—H⋯F hydrogen bonding interactions between the –NH_3_^+^ group and the BF_4_^−^ anions. The strongest of these are approximately parallel to the *a* axis and are 1.95 and 2.03 Å, for the two BF_4_^−^ orientations. Additional longer interactions (∼2.3 Å) are present between aniliniums and BF_4_^−^ within the same *bc* plane.

### 3.3. Crystal structure of polymorph B

AnBF_4_ is stable as polymorph B between ∼341 and ∼403 K and the structure was solved at 360 K using SXRD. This revealed a structure in *P*2_1_2_1_2_1_ with unit-cell parameters *a* = 7.3841 (3) Å, *b* = 6.1477 (3) Å and *c* = 18.4441 (8) Å (unit-cell parameters *a* and *b* are swapped from the standard setting to remain consistent with the other polymorphs). The cell is similar to that of polymorph A but with a doubled *c* axis. This monoclinic–orthorhombic phase transition is consistent with the convergence of the *hkl* and *hk**l* reflections, as anticipated from PXRD. The structure of polymorph B is similar to that of polymorph A, however alternating aniliniums in each chain displace along the *a* axis which leads to the cell doubling. Evidence for the cell doubling can be seen in Fig. 2 ▸ where ‘extra’ peaks (labelled *) appear at 11.3° (201) and 13.1° (211) in the temperature range 341–403 K, evidencing the loss of translational symmetry compared to polymorph A. The monoclinic angle β changes abruptly from 95.4° to 90° at the transition temperature (see the inset of Fig. 3 ▸), further evidence for the discontinuous nature of the transition. The BF_4_^−^ anions become disordered across two orientations in a 0.75:0.25 ratio in polymorph B. The shortest hydrogen bonds are slightly longer than those in polymorph A (2.03, 2.09 Å for the two BF_4_^−^ orientations).

### 3.4. Crystal structure of polymorph D

SXRD data could not be collected for the remaining two polymorphs due to the loss of integrity of the single crystal, so their structures were solved from PXRD data. Polymorph D was studied first as it is observed at the highest temperature, has the fewest Bragg peaks and is presumably the highest symmetry polymorph. The structure was solved using powder data recorded over 10.5 h at 477 K.

Based on the systematic absences in the powder pattern, space group *Pmmn* was chosen. This accounted for all peaks and a Pawley fit gave an *R*_wp_ of 4.5%. Refined unit-cell parameters were: *a* = 7.3115 (5) Å, *b* = 6.7187 (5) Å, *c* = 8.9102 (9) Å.

**Figure 5:**
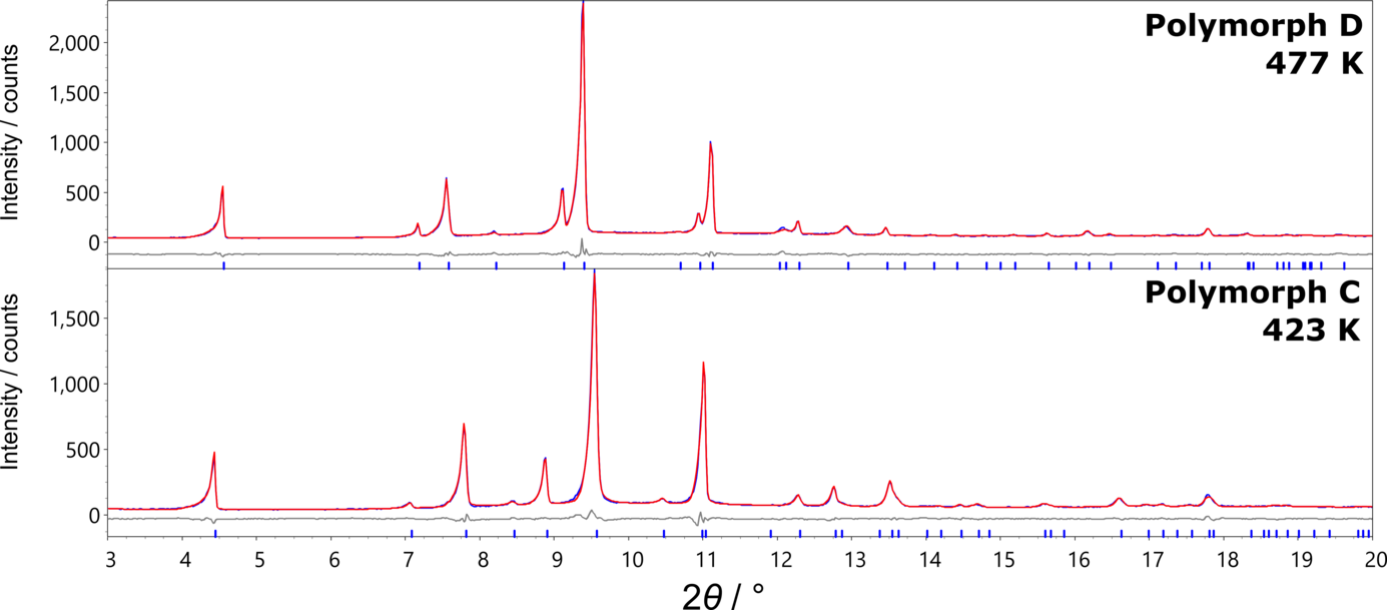
Powder X-ray diffraction data (blue) of AnBF_4_ showing the calculated patterns from Rietveld refinement (red) and the difference curve (grey) for polymorph C at 435 K and D at 477 K.

The structure was solved from the PXRD data by simulated annealing. A rigid body of protonated aniline was positioned at (0.5, 0.5, *z*) with the N—C bond parallel to *c*. Rotation about, and translation along the *c* axis were allowed to refine. The NH_3_^+^ group was allowed to disorder across two sites in line with the space-group symmetry. BF_4_^−^ ions were modelled using two tetrahedral rigid bodies with refinable bond lengths which generate 16 orientations by symmetry. Rigid body rotations and translations were allowed to randomize and refine for 100000 least-squares iterations. The best structure obtained was used in a Rietveld refinement giving an *R*_wp_ of 4.41% and the fit shown in Fig. 5 ▸. Refinement details are given in Table S2 and a powderCIF file is given in the supporting information. The anilinium molecules rotate such that they are at a similar angle to the *bc* plane as they are in polymorph A (25°), but are disordered across two orientations in a 50:50 ratio. The 16 potential orientations of the BF_4_^−^ ions when viewed together show little anisotropy suggesting the BF_4_^−^ anions are freely rotating in this phase.

### 3.5. Crystal structure of polymorph C

During SXRD analysis, preliminary unit-cell dimensions were recorded before crystal breakdown in the temperature region of polymorph C (435 K), giving an orthorhombic cell [*a* = 7.362 (8) Å, *b* = 6.572 (7) Å and *c* = 9.012 (9) Å]. The unit-cell parameters are similar to those of polymorphs A and D, but indicate that the cell doubling seen in B has been lost. The structure of polymorph C was solved from PXRD data recorded over 11 h at 423 K. The clearest distinction between the diffraction patterns of D and C is the presence of the (210) reflection in the latter. This suggests that the *n*-glide present in D is not present in C. Space group *P*2_1_2_1_2 fits this observation and is both a maximal non-isomorphic *translationengleiche* subgroup of *Pmmn* (D) and a maximal non-isomorphic *klassengleiche* supergroup of *P*2_1_2_1_2_1_ (**c**′ = 2**c**; hence the cell doubling seen in B).

The loss of symmetry between polymorphs D and C can be achieved by removing the disorder in the anilinium rings. The C—NH_3_^+^ bond remains collinear with the twofold rotation axis of *P*2_1_2_1_2. A model based on these assumptions achieved an *R*_wp_ of 4.69% with unit-cell parameters *a* = 6.3269 (5) Å, *b* = 7.3729 (5) Å and *c* = 9.1350 (10) Å. The resulting Rietveld refinement is shown in Fig. 5 ▸ and the structural model is shown in Fig. 4 ▸(*b*). Due to the reduction in symmetry, the number of orientations of BF_4_^−^ decreases from 16 to 8. Unlike in polymorph D, the BF_4_^−^ groups seem to display orientational preferences. One threefold axis in each points approximately along the *c* axis similar to the orientations seen in polymorphs B and A.

Due to the significant disorder in the two phases solved from powder data, less concrete conclusions can be drawn about the hydrogen bonding in polymorphs C and D. As the strongest hydrogen bonding interactions point along the *a* axis in polymorphs A and B, the low thermal expansion of the *a* axis in the entire temperature range studied might imply that these interactions are still important within polymorphs C and D.

The competing energetics between this and the entropy stabilization gained by the facile BF_4_^−^ rotations may rationalize the large number of polymorphs observed within the studied temperature range.

### 3.6. Diffuse scatter in polymorph C

**Figure 6:**
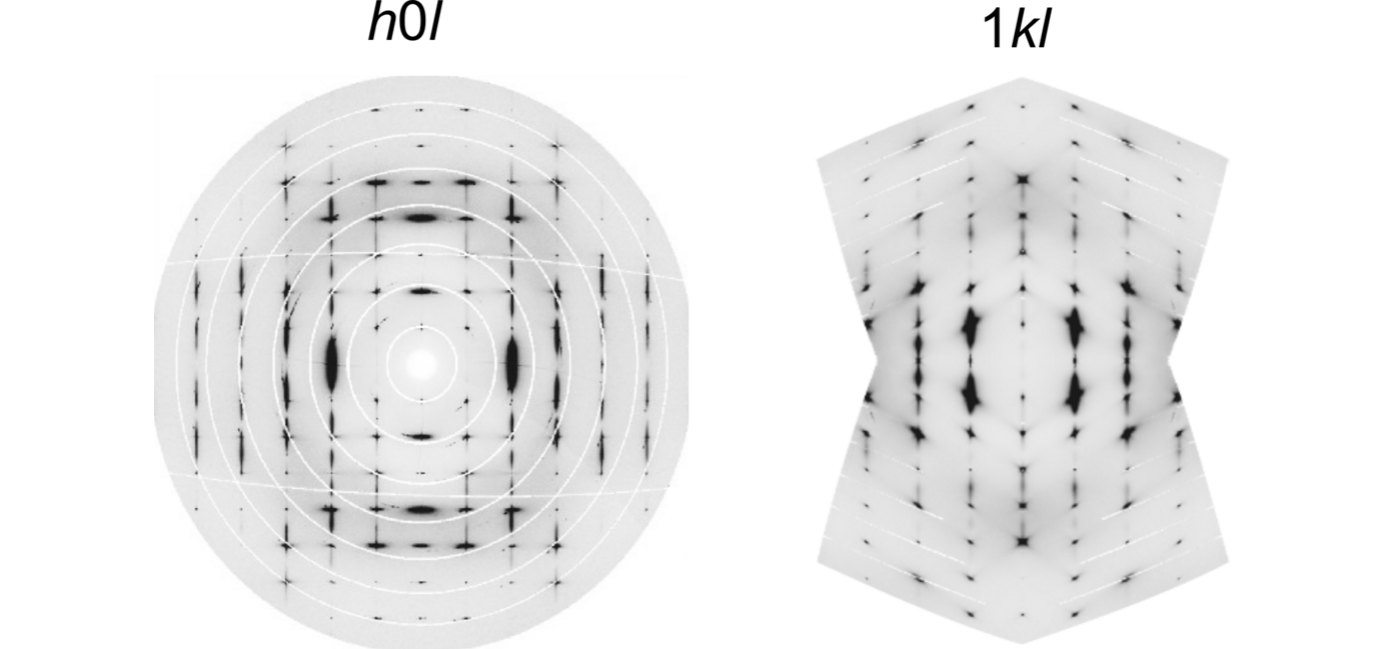
Reconstructed experimental *h*0*l* and 1*kl* scattering planes of polymorph C at 430 K. c* runs up the page in both images.

Synchrotron single crystal X-ray diffraction experiments were also performed on a small crystal of AnBF_4_ which remained intact to 430 K where C is the stable polymorph. The average structure was consistent with that derived from PXRD, but significant diffuse scattering was seen between the Bragg peaks, suggesting short range structural correlations. This was most prominent in the *h*0*l* and 1*kl* planes shown in Fig. 6 ▸ and predominantly appears as rods underneath the Bragg peaks along the *l* direction. This implies the presence of a stacking-like disorder, where long-range order is preserved in the *ab* plane but there is disorder between the planes in the [001] direction. Simple models to facilitate the qualitative interpretation of the diffuse scatter were developed by considering the anilinium order–disorder processes present in the adjacent high-temperature polymorphs B and D. These are detailed in the supporting information. We find that short-range correlated displacement of molecules similar to that present in the ordered polymorph B [Fig. 4 ▸(*b*)] contributes to the diffuse scatter. As can be seen in Fig. S3, some experimental features are not captured by this simple model, notably the crescent shapes appearing at the top and bottom of the *h*0*l* image and discrepancies around the 400 and 110 reflections. This is likely due to the model neglecting any accompanying short-range order of the BF_4_^−^ ions.

### 3.7. Solid-state NMR

**Figure 7:**
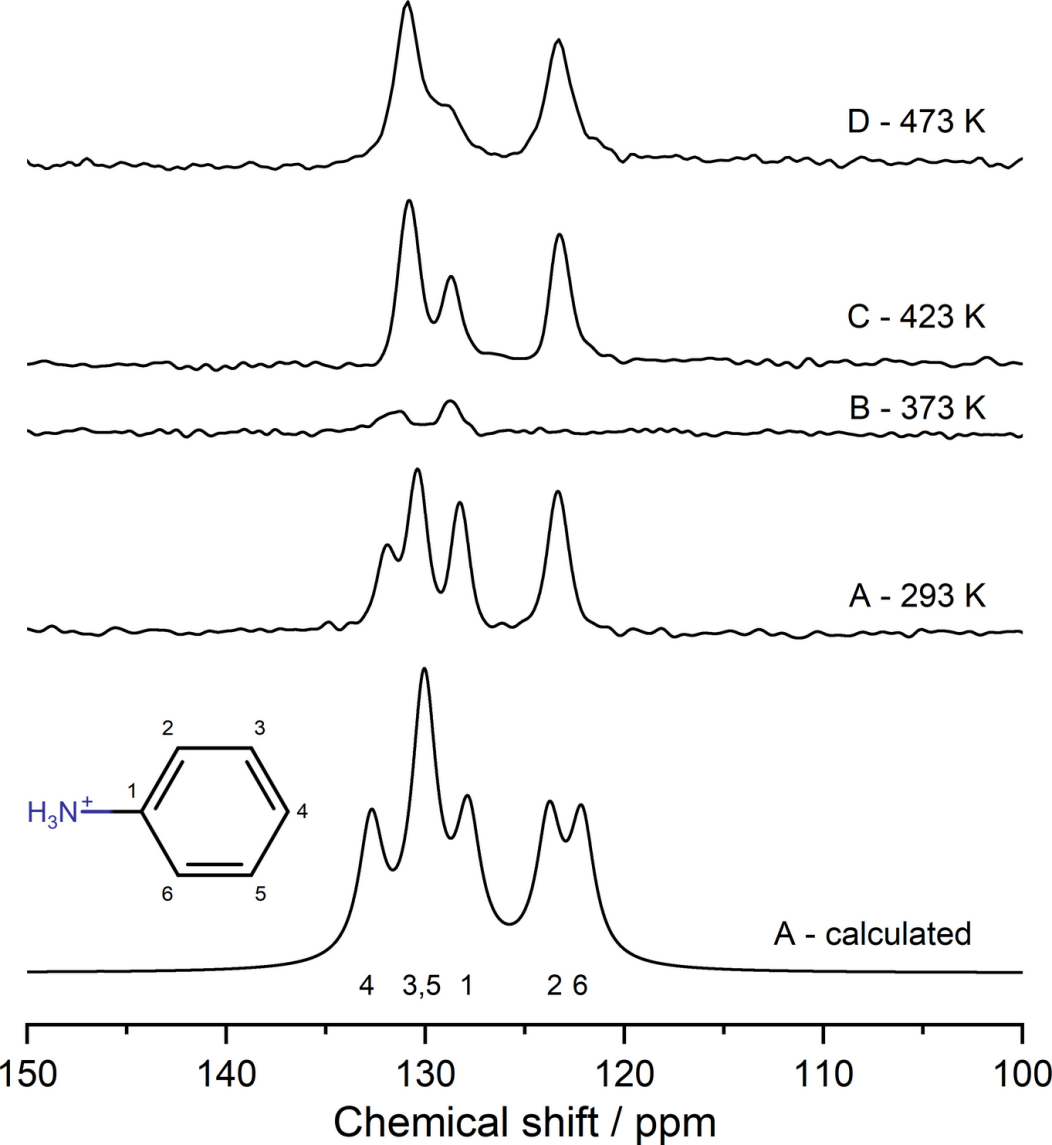
Calculated and experimental ^13^C solid-state NMR CPMAS spectra acquired at 100.63 MHz and a 20 kHz MAS rate.

Solid-state ^13^C NMR data were collected for each AnBF_4_ polymorph. At 293 K, four distinct peaks are observed. To assign these, chemical shifts were predicted using DFT in *CASTEP*. After referencing, the peaks were assigned as shown in Fig. 7 ▸, which reveals good general agreement between calculated and observed spectra. Experimentally, sites 2 and 6 overlap, leading to a single observed peak.

Upon warming to 373 K (the temperature region of polymorph B), a significant loss in intensity is observed for all peaks suggesting activation of a dynamic motion around the twofold symmetry axis with a timescale on the order of 20–83 kHz for the carbon atoms.

At 423 K, in the temperature region of polymorph C, similar overall intensity is observed to that at 298 K suggesting the dynamic feature in polymorph B moves to higher frequency. Carbon atoms 3, 4 and 5 become indistinguishable at the experimental resolution. Further heating to 473 K (polymorph D), leads to minor peak shifts and broadening.

^19^F solid-state NMR spectra were also recorded on each polymorph to investigate the static/dynamic disorder of the BF_4_^−^ ions. A single resonance was observed for each phase (Fig. S2) suggesting either that sites are indistinguishable at the experimental resolution, or that rapid exchange occurs on the NMR timescale in all polymorphs.

### 3.8. Dielectric properties

**Figure 8:**
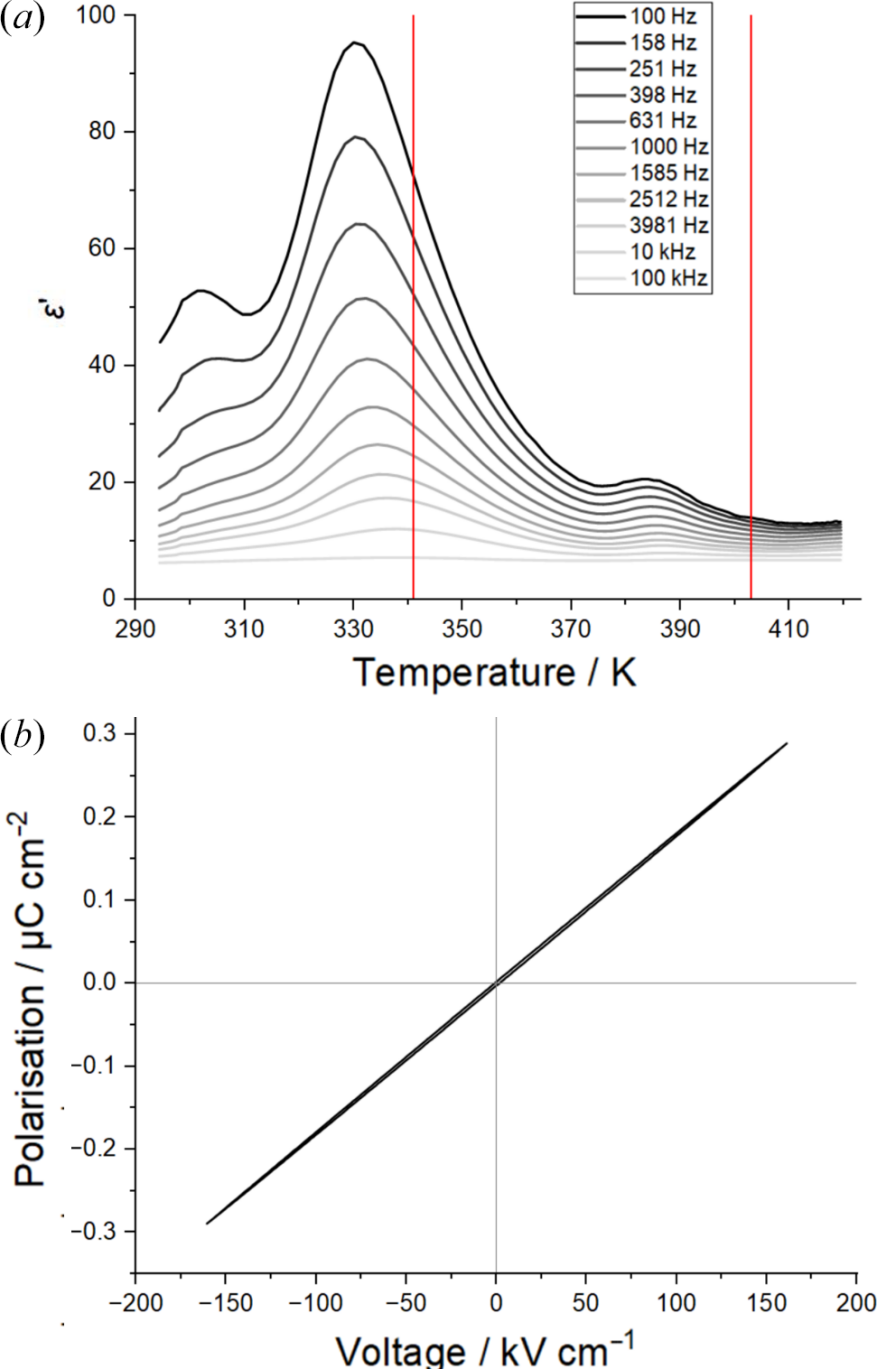
(*a*) The real part of the dielectric constant of AnBF_4_ measured between 293 and 420 K. (*b*) Polarization–electric field data measured on a pressed pellet of AnBF_4_ at 293 K with a switching frequency of 100 Hz.

The dielectric constant of a pressed pellet of AnBF_4_ was measured at frequencies between 100 Hz and 100 kHz as the temperature was increased from 293 K to 420 K [Fig. 8 ▸(*a*)]. The transition from A to B is observed as a smooth Gaussian-like peak in the real dielectric constant (ɛ′) at 333 K. This occurs close to the phase transition temperature of 341 K determined by VT-PXRD. The smooth peak observed in this experiment differs from the expected lambda-point features observed at *T*_C_ in conventional ferroelectrics. This is likely due to the fact that phase B is not the true paraelectric phase so the rapid dipole switching that causes the sharp peak in conventional ferroelectrics does not occur. Of course, it could also be evidence that polymorph A is not a ferroelectric material. A second peak is observed at 387 K, slightly below the PXRD measured temperature of 403 K for the transition from polymorph B to C. The experimental set-up used was not able to reach the temperature required for the transition from C to D (expected at 454 K).

A fresh pellet of AnBF_4_ was used to measure its polarization response in an electric field at room temperature. With a switching frequency of 100 Hz, AnBF_4_ showed good dielectric behaviour beyond fields of 100 kV cm^−1^. The switching frequency was reduced in case 100 Hz exceeded the maximum intrinsic switching frequency of the structure, however hysteretic behaviour indicating ferroelectricity could not be observed at room temperature.

### 3.9. Structural relationship between polymorphs

**Figure 9:**
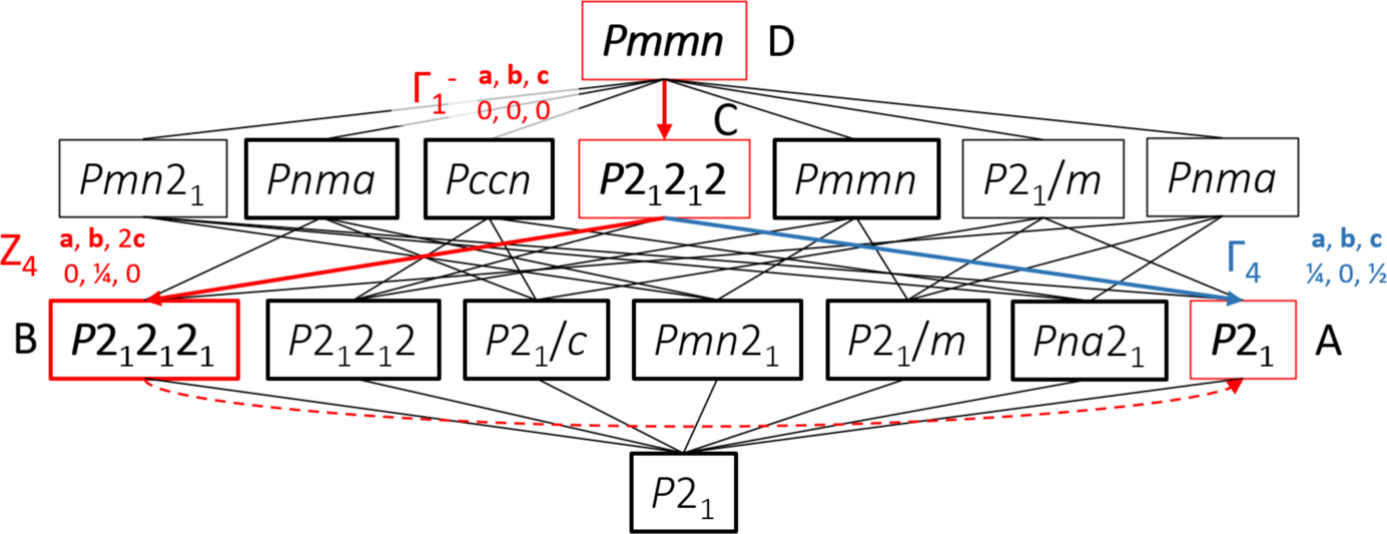
Group–subgroup tree containing all known polymorphs of AnBF_4_ from the high-symmetry parent structure D to a hypothetical structure with doubled *c* axis in *P*2_1_. Red boxes indicate observed structures and bold boxes indicate a doubled parent *c* axis. Red transitions are experimentally observed with dashed lines indicating transitions between groups with no direct group–subgroup relationship. Important transitions are labelled with their irrep labels, cell transformation and origin shift.

The similarity in the diffraction patterns of the observed phases of AnBF_4_ suggests a close structural relationship between them that can be described using the language of isotropy subgroups (Stokes *et al.*, 2024 ▸). A group–subgroup tree drawn using the parent group *Pmmn* of D and a child base subgroup (Lewis *et al.*, 2016 ▸) *P*2_1_ with doubled *c* axis is shown in Fig. 9 ▸. The molecular arrangements for all four polymorphs are generally similar and are described above for polymorph A.

The transition from polymorph D to C that occurs at ∼461 K can be described by the superposition of Γ_1_^−^ occupancy modes on the disordered anilinium molecules of the *Pmmn* parent structure. The basic structure is essentially unchanged across the transition with little displacement occurring for either the cation or the anion. The main difference is the ordering of the anilinium ions from two orientations to one, which removes the symmetry planes of the *Pmmn* space group. The resulting structure C has neighbouring anilinium chains (along the [110] and [110] directions) rotated in an alternating clockwise/anticlockwise sense [Fig. 4 ▸(*b*)]. Our model suggests a partial ordering for the BF_4_^−^ ion from eight orientations to four. In Landau theory, a continuous phase transition generally requires that the symmetry breaking be described by a single irreducible representation (irrep); multi-irrep transitions are always first-order, while single-irrep transitions may be continuous depending on the allowed invariants in the free energy.

The single irrep that is active and the smooth evolution of unit-cell parameters and hence Bragg peak positions at 461 K seen in Fig. 2 ▸ and Fig. 3 ▸ are consistent with DSC data indicating weakly first-order behaviour.

Similarly, the transition from polymorph C to B can be described by the superposition of *Z*_4_ displacement modes on the structure of C. These modulate the structure such that alternate anilinium molecules are displaced relative to one another along the *a* direction. While the transition is allowed to be continuous and most strong Bragg peaks evolve smoothly across the transition, DSC data show a small latent heat consistent with the abrupt appearance of superstructure reflections. B can also be described directly as by the superposition of two irreps on D.

The transition from B to A is discontinuous as shown by the sharp changes in the diffraction pattern at ∼341 K. From the subgroup tree of Fig. 9 ▸, there is no single irrep (no single line) linking B to A and thus the transition must be discontinuous. The transition simultaneously features the loss of the displacement that arose at the C to B transition and development of structural and strain modes transforming as the irrep Γ_4_ which together relate C to A (creating the monoclinic cell from an orthorhombic one). The significant structural changes are consistent with the large hysteresis in transition temperatures on warming and cooling.

## 4. Conclusions

Anilinium tetra­fluoro­borate exhibits three symmetry-raising phase transitions on heating that have been characterized by variable-temperature diffraction, symmetry analysis, and dielectric measurements. Starting from a polar monoclinic structure at room temperature, the crystal undergoes a discontinuous transition to an orthorhombic form (*P*2_1_2_1_2_1_, polymorph B) which leads to changes in the dielectric data. This is followed by two weakly discontinuous transitions leading to higher-symmetry polymorphs C (*P*2_1_2_1_2) and D (*Pmmn*). These transitions are driven by molecular displacement (A→B) and order–disorder (B→C→D) of both the cation and the BF_4_^−^ anion. The symmetry changes can be rationalized using a group–subgroup tree and irreducible representation analysis. The specific structural transition to space group *P*2_1_/*m* predicted by *FERROSCOPE* was not observed with changing temperature, but changing a different thermodynamic variable such as pressure studies may reveal this and other polymorphs (Jiang *et al.*, 2024 ▸; Szafranski, 2014 ▸). Despite this, the overall structural changes predicted were observed, namely, the rotational disorder of BF_4_^−^ and the mirror symmetry gained between anilinium rings. This demonstrates the ability of our symmetry-raising prediction method.

## Supplementary Material

Crystal structure: contains datablock(s) global, qehweg_a, qehweg_b, C_1, d6b_01628_415K.raw_1, D_1, d6b_01628_480K.raw_1. DOI: 10.1107/S2052520625009618/ra5160sup1.cif

Structure factors: contains datablock(s) qehweg_a. DOI: 10.1107/S2052520625009618/ra5160qehweg_asup2.hkl

Structure factors: contains datablock(s) qehweg_b. DOI: 10.1107/S2052520625009618/ra5160qehweg_bsup3.hkl

Raw phase C PXRD data. DOI: 10.1107/S2052520625009618/ra5160sup4.bin

Raw phase D PXRD data. DOI: 10.1107/S2052520625009618/ra5160sup5.bin

Variable temperature PXRD data. DOI: 10.1107/S2052520625009618/ra5160sup6.bin

Section S1, Tables S1-S1 Figs. S1-S3. DOI: 10.1107/S2052520625009618/ra5160sup7.pdf

Supporting information file. DOI: 10.1107/S2052520625009618/ra5160qehweg_asup8.cml

CCDC references: 2403155, 2403156, 2499478, 2499479
