# Supplementary material for: Prediction and analysis of symmetry-raising transitions in anilinium tetra­fluoro­borate
Source: Acta Crystallogr B Struct Sci Cryst Eng Mater. 2025 Nov 13;81(Pt 6):587–94. doi: 10.1107/S2052520625009618 (PMC12786386; doi:10.1107/S2052520625009618)
Supplement: Supplementary file 7 [file b-81-00587-sup7.pdf]

STRUCTURAL SCIENCE
CRYSTAL ENGINEERING
MATERIALS

Volume 81 (2025)

Supporting information for article:

Prediction and analysis of symmetry-raising transitions in anilinium tetrafluoroborate

Sam Y. Thompson, Chloe A. Fuller, Samuel J. Page, Andrew J. Bell and John S. O. Evans

Table S1 SXRD experimental and refinement details.

	Polymorph A	Polymorph B
Crystal data		
Chemical formula	$BF_4 \cdot C_6H_8N$	$BF_4 \cdot C_6H_8N$
M_r	180.94	180.94
Crystal system, space group	Monoclinic, $P2_1$	Orthorhombic, $P2_12_12_1$
Temperature (K)	290	360
a, b, c (Å)	7.3672 (9), 5.9633 (7), 9.3181 (11)	6.1477 (3), 7.3841 (3), 18.4441 (8)
β (°)	96.520 (4)	-
V (Å^3)	406.72 (8)	837.27 (6)
Z	2	4
Radiation type	Mo $K\alpha$	Mo $K\alpha$
μ (mm^{-1})	0.15	0.15
Crystal size (mm)	0.22 × 0.13 × 0.07	0.22 × 0.13 × 0.07
Data collection		
Diffractometer	Bruker D8 Venture	Bruker D8 Venture
T_{min}, T_{max}	0.630, 0.746	0.657, 0.746
No. of measured, independent and observed [$I > 2\sigma(I)$] reflections	1718, 1710, 1145	15570, 2787, 1115
R_{int}	0.050	0.053
$(\sin \theta/\lambda)_{max}$ (Å^{-1})	0.638	0.737
Refinement		
$R[F^2 > 2\sigma(F^2)]$, $wR(F^2)$, S	0.058, 0.175, 1.08	0.099, 0.335, 1.03
No. of reflections	1710	2787
No. of parameters	148	156
$\Delta\rho_{max}$, $\Delta\rho_{min}$ (e Å^{-3})	0.46, −0.30	0.31, −0.22
Absolute structure parameter	−0.8 (10)	−0.1 (4)

Table S2 PXRD experimental and refinement details

	Polymorph C	Polymorph D
Empirical formula	$BF_4 \cdot C_6H_8N$	$BF_4 \cdot C_6H_8N$
Formula weight	180.94	180.94

Space group	$P2_12_12_1$	*Pmmn*
Unit cell dimensions		
a / Å	7.3727(5)	7.3113(4)
b / Å	6.3267(5)	6.7173(5)
c / Å	9.1348(10)	8.9094(7)
β / °		
V / Å^3	426.09(7)	437.56(5)
Z	2	2
D_x / g cm^{-3}	1.4103(3)	1.37333(16)
Radiation	Mo $K\alpha$	Mo $K\alpha$
2θ / °	3−30	3−30
T / K	423	477
B_{eq} / Å^2	9.0(3)	12.4(3)
R_{wp} (%)	4.58	4.42
R_{Bragg} (%)	1.92	1.77
R_p (%)	3.40	3.46
GOF	2.10	1.98

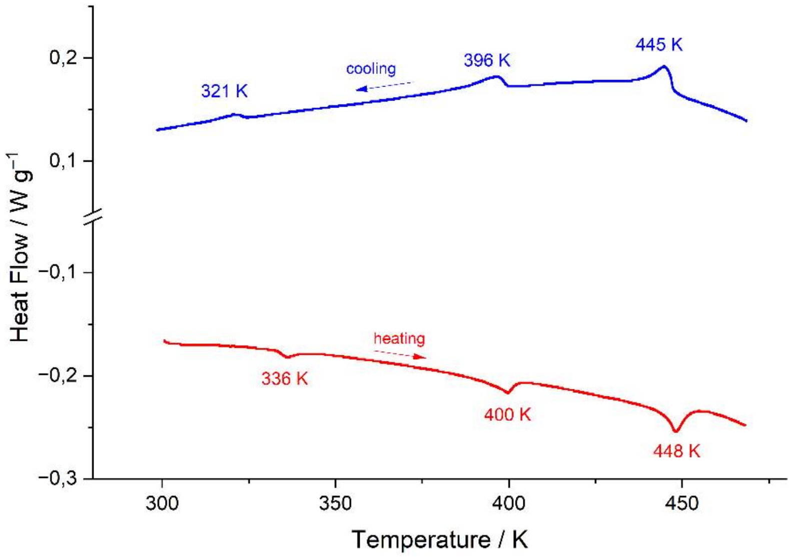

Figure S1 Differential scanning calorimetry measured on anilinium tetrafluoroborate. The transitions were observed repeatedly on multiple heating/cooling cycles.

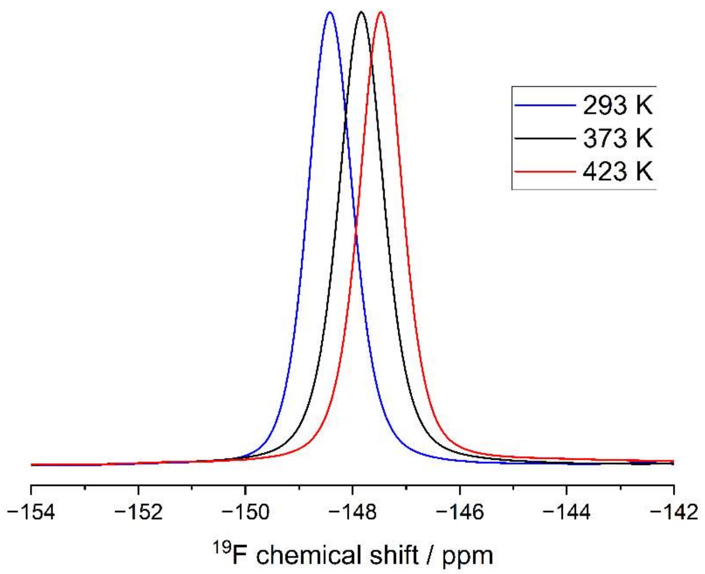

Figure S2 ^{19}F NMR MAS spectra acquired at 376.50 MHz and a 20 kHz MAS rate.

S1. Diffuse scattering DISCUS simulations

To investigate the origin of the diffuse scatter in polymorph C, two models were generated derived from the molecular positioning of the aniliniums in polymorphs B and D. One model was based on the anilinium displacements causing the cell doubling of polymorph B producing "up" and "down" displacive sites. A second model was based on the rotational disorder that arises in the rings during the transition from C to D producing disordered 'clockwise' and 'anticlockwise' sites.

To test each case, a $32\times32\times32$ supercell of anilinium rings was created from the unit cell of polymorph C in DISCUS (Proffen & Neder, 1997). Disorder was introduced into the model by randomly changing the coordinates of half of the anilinium rings (to either a translated or rotated position). Relationships between neighbouring rings were established, as illustrated in the figure below, with the interaction parameter, k_{NN} describing the strength of the interaction between the neighbours. In this simple model, only the nearest neighbours of the rings were considered, and the disorder in the BF_4^- ions was neglected.

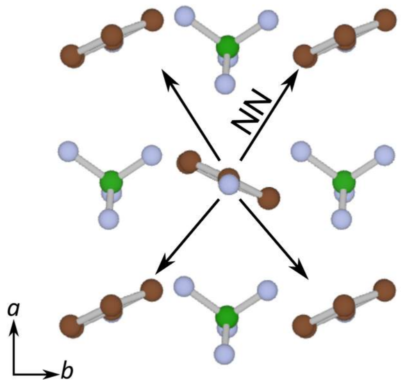

The disordered rings were locally ordered by a Monte Carlo algorithm using the following Ising energy term as the Hamiltonian:

$$E = \sum_{\langle ij \rangle} k_{NN} \sigma_i \sigma_j,$$

where $\langle ij \rangle$ indicates that each pair of sites *i* and *j* are only counted once, and σ_i is an Ising variable (either +1 or -1) representing the “up” or “down” (“clockwise” or “anticlockwise”) sites. MC moves are accepted if the energy of the system decreases following a move. If not, it is accepted with a probability proportional to

$$\exp\left(-\frac{\Delta E}{kT}\right)$$

For the simulations in the work $T = 1.0$ was used, and k_{NN} was chosen to be −0.8 such that the rings of like orientations will prefer to cluster together. The magnitude of these are arbitrary choices and were found by trial and error to give a reasonable qualitative match to the experimental diffuse scattering (although a thorough search of the parameter space was not undertaken).

Following the construction of the locally ordered model, the resulting diffuse scattering in the *h*0*l* and 1*kl* planes were calculated using the following parameters.

Calculation method	LOTS. 100*(20x20x20) Eliptical
Average subtraction	Yes, calculated from 100% of crystal
*h*0*l*	
x-axis	$-6 \le h < 6$, 241 pixels
y-axis	$-8 \le l < 8$, 321 pixels
1*kl*	
x-axis	$-4 \le k < 4$, 241 pixels
y-axis	$-8 \le l < 8$, 481 pixels

Calculated diffuse scattering in the *h*0*l* and *1kl* planes for both the translational and rotational disorder models are shown in Figure S3. Despite being very minimal, the translational disorder model effectively captures many of the prominent diffuse features, and the positions of the maxima in the diffuse rods along *l* are accurately reproduced in both scattering planes. The rotational model, on the other hand, produces no significant diffuse scattering in *h*0*l* and unobserved maxima in 1*kl*.

This interpretation is consistent with the observed structural changes in each polymorph of $AnBF_4$: the translational disorder that becomes frozen-in in B appears to be retained over short length scales in C, but the rotational disorder is not thermally accessible until the transition to D. We note that some experimental features are not captured by this simple model, notably the crescent shapes appearing at

the top and bottom of the *h*0*l* image. This is likely due to the model neglecting any accompanying short-range order of the BF_4^- ions.

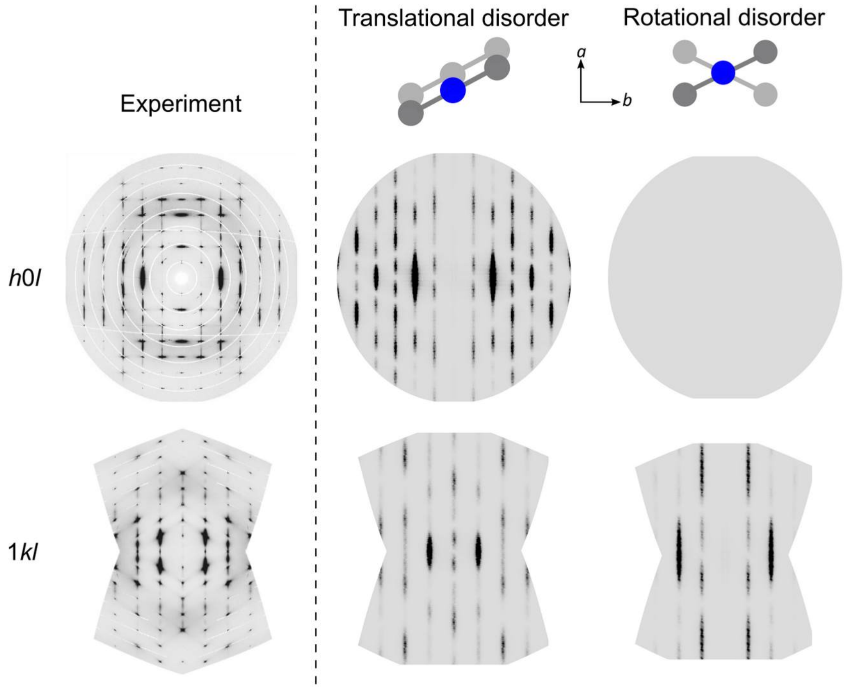

Figure S3 Left: Reconstructed experimental *h*0*l* and 1*kl* scattering planes of polymorph C at 430K. Centre/Right: Simulated planes in which the Bragg diffraction has been removed leaving only the diffuse scattering. The two schematics show the anilinium disorder model in the two simulations. The view direction is that of Error: Reference source not foundb.
